# Tabu search based global optimization algorithms for problems in computational chemistry

**DOI:** 10.1186/1758-2946-4-S1-P10

**Published:** 2012-05-01

**Authors:** Christoph Grebner, Johannes Becker, Daniel Weber, Bernd Engels

**Affiliations:** 1 Institute of Physical and Theoretical Chemistry, University of Wuerzburg, Emil Fischer Strasse 42, Wuerzburg, 97074, Germany

## 

Efficient searches for global minima of highly dimensional functions with numerous local minima are central for the solution of many problems in computational chemistry. Well known examples are the identification of the most stable conformer of molecules possessing a high number of freely rotatable bonds [1] or the equilibration phase for QM/MM computations. Mathematically, both represent global optimization problems in which the potential energy function of the molecule is the objective function while the coordinates used for representing the structural arrangement of the system are the variables.

Based on an analysis of well-known metaheuristic algorithms, several new global optimization algorithms based on Tabu Search (TS) were developed in our group [2,3], which are using a *steepest descent – modest ascent* strategy. In a first application the Gradient Only Tabu Search (GOTS) was shown to be applicable to conformational search problems [4].

Further test calculations showed its high efficiency in comparison to other global search algorithms like Molecular Dynamics, Simulated Annealing or Monte Carlo Minimization (MCM). The tests also revealed that the efficiency of GOTS can be enhanced dramatically by combining GOTS (searches the nearest neighbourhood highly efficient) with short MCM simulations (samples the phase space more widely) [5]. The new algorithm is implemented into a program for conformational search, providing several different force fields and sampling algorithms.

The investigations also pointed out that a key point is the algorithm for the *modest ascent* strategy. For providing a smoother scan of the potential energy surface as well as a more accurate description of the minimum energy path between two found minima, the Dimer-Method for transition state search was implemented into GOTS-MCM. First tests show that it is much more efficient than previous versions of GOTS. The new algorithm is applied to solvation of biomolecules to provide global optimized solvent shells [6].

In future, the GOTS algorithm will also be used for a proper prediction of reaction pathways by global optimized minimum energy paths and molecular modelling or docking of potential drug molecules to enzymes.
